# Restoring E-cadherin Expression by Natural Compounds for Anticancer Therapies in Genital and Urinary Cancers

**DOI:** 10.1016/j.omto.2019.04.005

**Published:** 2019-05-17

**Authors:** Yizuo Song, Miaomiao Ye, Junhan Zhou, Zhi-wei Wang, Xueqiong Zhu

**Affiliations:** 1 Departmant of Obstetrics and Gynecology, The Second Affiliated Hospital of Wenzhou Medical University, Wenzhou, Zhejiang 325027, China; 2 Department of Pathology, Beth Israel Deaconess Medical Center, Harvard Medical School, Boston, MA, USA

**Keywords:** E-cadherin, EMT, invasion, cancer, natural agents, therapy

## Abstract

E-cadherin plays a pivotal role in cancer progression, including the epithelial-mesenchymal transition (EMT) process and tumor metastasis. Loss of E-cadherin contributes to enhanced invasion and metastasis in human cancers. Therefore, restoring E-cadherin could be a potential approach for cancer therapy. Multiple natural compounds have been shown to possess anti-tumor activities through the regulation of key molecules in signaling pathways, including E-cadherin. In this review, we describe the numerous compounds that restore the expression of E-cadherin in genital and urinary malignancies. We further discuss the potential anti-tumor molecular mechanisms of these agents as the activators of E-cadherin in genital and urinary cancers. Although these compounds exhibit their potential to inhibit the development and progression of cancers, there are several challenges to developing them as therapeutic drugs for cancer patients. Poor bioavailability *in vivo* is the main disadvantage of these compounds. Modification of compound structures has produced actual improvements in bioavailability. Nanoparticle-based delivery systems could be useful to deliver the agents to targeted organs. These compounds could be new promising therapeutic agents for the treatment of human genital and urinary cancers. Further investigations are required to determine the safety and side effects of natural compounds using animal models prior to clinical trials.

## Main Text

Cell-cell adhesion plays a key role in morphogenetic processes in development and tissue organization. It contributes to the determination of cell shape, polarity, and tissue integrity, which are involved in cell differentiation.^1^, ^2^ Adherens junctions (AJs) are specialized intercellular structures that link cells together and regulate cytoskeleton reorganization, intracellular signaling, and transcriptional regulation. Moreover, AJs involve calcium-dependent cell-cell adhesion molecules of the cadherin family and their associated catenins.^3^, ^4^ Cadherins play important roles in cell adhesion, and E-cadherin is a key prototype of this superfamily that is expressed by epithelial cells.^5^

**Figure 1:**
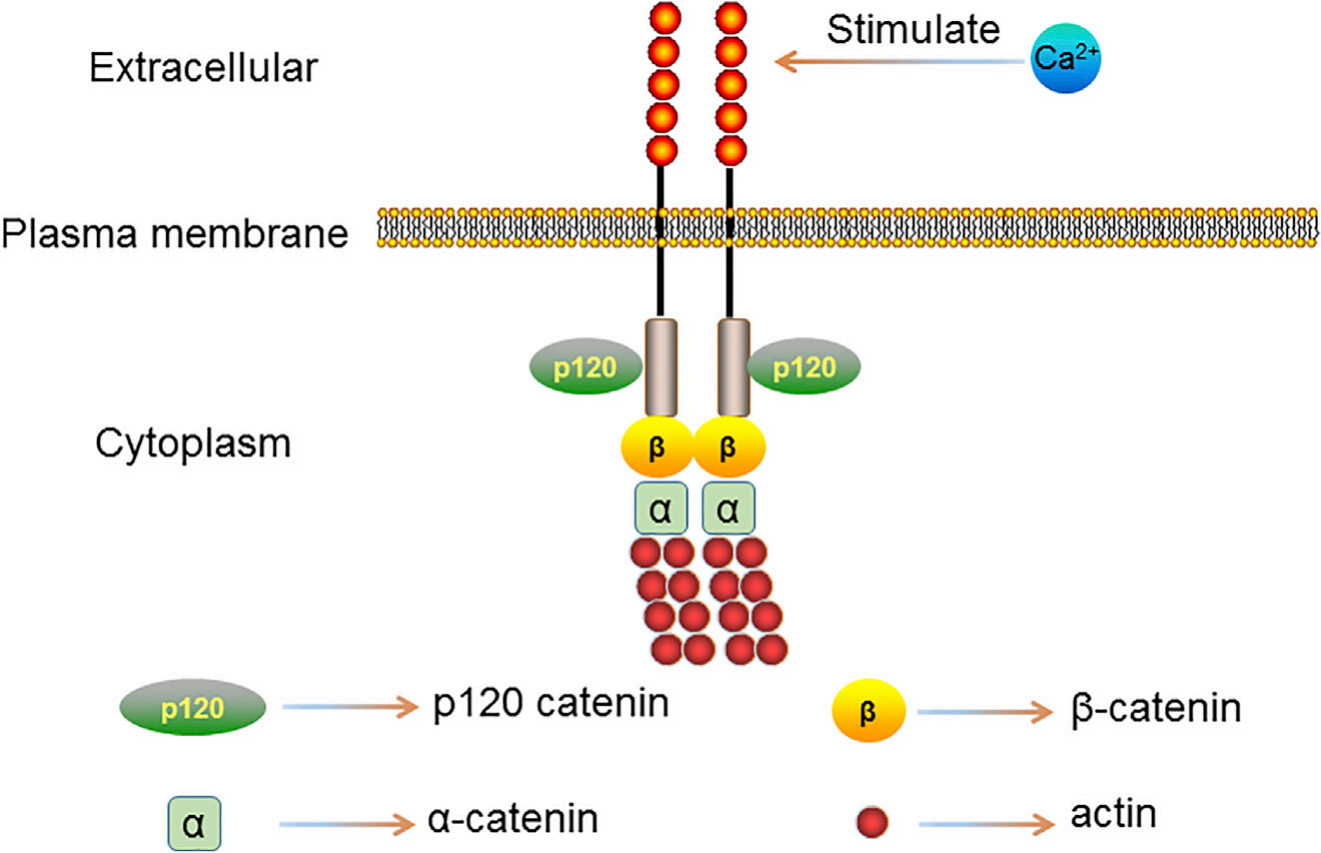
The Structure of E-cadherin E-cadherin is a transmembrane glycoprotein consisting of five calcium-dependent extracellular domains (EC1–EC5) that confer homotypic interactions on the surface of a neighboring cell, a transmembrane domain, and a cytoplasmic domain that binds to members of the catenin protein family to transduce physical and biochemical signals to the cell, including β-catenin and p120 catenin.

E-cadherin, encoded by CDH1 (16q22) gene, is a 120-kDa transmembrane glycoprotein consisting of five calcium-dependent extracellular domains (EC1–EC5) that confer homotypic interactions on the surface of a neighboring cell, a transmembrane domain, and a cytoplasmic domain that binds to members of the catenin protein family to transduce physical and biochemical signals to the cell, including β-catenin and p120 catenin.^3^, ^6^ The N terminus of β-catenin binds directly to the C-terminal catenin domain (CBD) of E-cadherin, while α-catenin interacts with the C terminus of β-catenin via its N-terminal domains, connecting AJs to the actin cytoskeleton, while p120 catenin binds to the juxtamembrane domain (JMD) and enhances the stability of E-cadherin by regulating the turnover of E-cadherin at the cell surface^7^, ^8^ (Figure 1).

**Figure 2:**
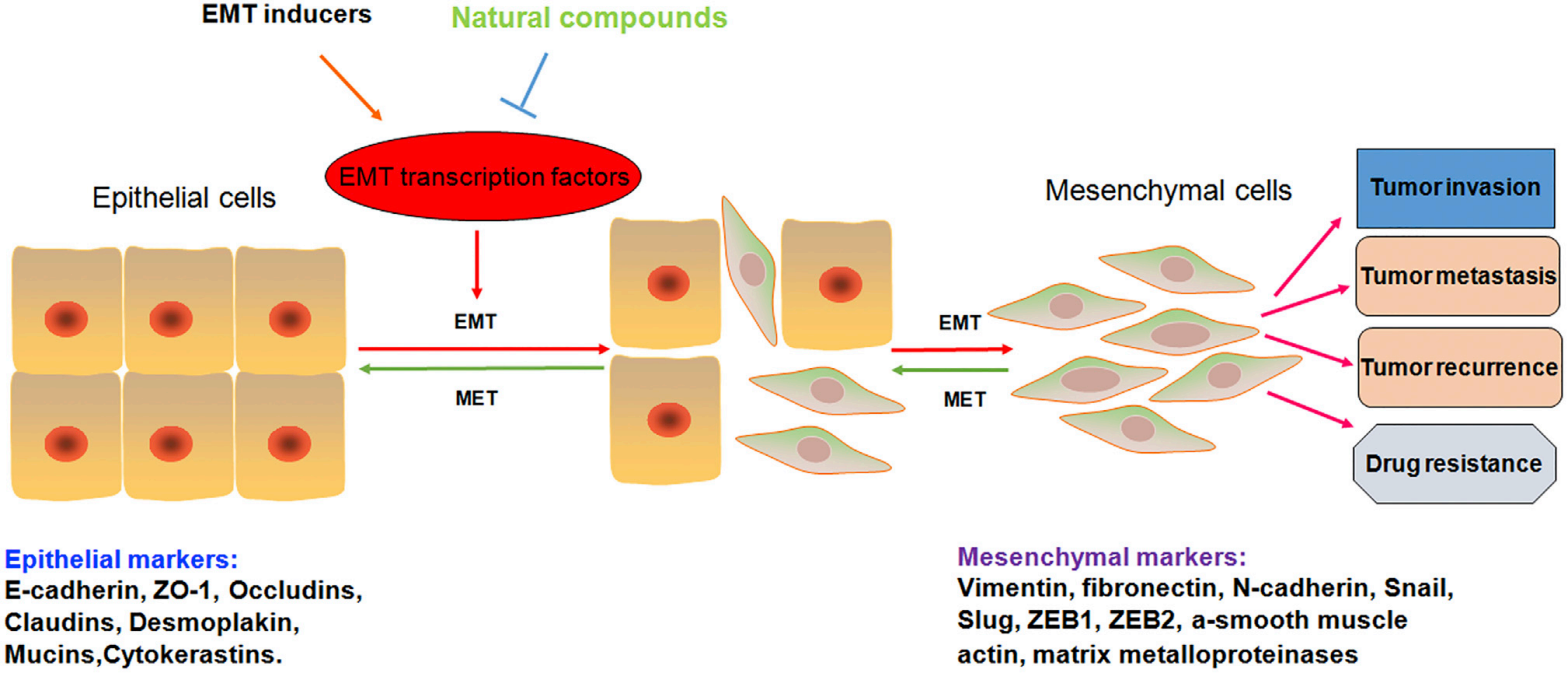
The EMT Process Enhances Tumor Invasion and Metastasis Epithelial cells become meshenchymal cells after the EMT inducers trigger. The tumor cells obtain increased invasion and metastasis, drug resistance, and tumor recurrence. Natural compounds could reverse the EMT process to the MET via upregulation of E-cadherin and inhibition of mesenchymal marker expression.

Normal cells inhibit their growth and migration when cells adhere to each other. However, these properties are progressively lost in tumor cells, contributing to increased rates of cell proliferation and migration. Not surprisingly, E-cadherin loss is relatively common in cancers of epithelial origin. Mounting evidence has revealed that the downregulation of E-cadherin results in less intercellular contact and reduced cell polarity, promoting the transformation of epithelial cells to mesenchymal stem cells, which is one of the hallmarks of the epithelial-mesenchymal transition (EMT), as seen in carcinogenesis and cancer cell invasion processes^9^, ^10^ (Figure 2).

The levels of E-cadherin are regulated by multiple processes, such as gene transcription, post-translational modification, and protein turnover at the membrane.^11^ E-cadherin transcription is directly regulated by methylation of promoter activity. Methylation is a common DNA modification mediated by a family of DNA methyltransferase enzymes that catalyze the addition of a methyl group to cytosine residues at CpG dinucleotides.^12^ In most cancers, E-cadherin expression is downregulated due to promoter methylation, causing the tumor progression and metastasis.^13^, ^14^, ^15^ Src tyrosine kinase-mediated phosphorylation, as a major post-translational mechanism of E-cadherin level, induces the ubiquitination of E-cadherin and EMT occurrence.^16^ Conversely and interestingly, phosphorylation of E-cadherin at serine residues within the CBD by glycogen synthase kinase-3 beta (GSK-3β) leads to a substantial increase in the affinity of E-cadherin for β-catenin.^17^ Numerous studies have suggested that a core function of p120 catenin is being able to regulate cadherin turnover. Normally, E-cadherin is rapidly endocytosed and degraded when it fails to interact with p120 catenin. Furthermore, interaction of p120 with E-cadherin increases the half-life of E-cadherin substantially, indicating that p120 is essential for improving adhesive strength by controlling the amount of E-cadherin available for adhesion.^8^

There is growing evidence that E-cadherin plays a crucial role in the invasion and metastasis of human cancer, including genital and urinary cancers, indicating that E-cadherin could be a promising therapeutic target for designing anti-tumor drugs. Therefore, we mainly reviewed the studies focusing on pharmacological compounds targeting E-cadherin in the treatment of genital and urinary cancers, which have emerged in the last 5 years.

### Compounds Increase the E-cadherin Expression in Genital and Urinary Cancers

Numerous efforts have been made to discover potent compounds that influence cellular E-cadherin level and strengthen cell-cell adhesions in cancer cells. However, it is noteworthy that, for the majority of these compounds, detailed molecular mechanisms of their actions on cancer cells remain to be elucidated. In the following sections, a number of extracted or synthetic molecules have been identified to restore the expression of E-cadherin, which eventually inhibits the cell invasion and migration in human genital and urinary malignancies (Table 1).

**Table 1:** Compounds that Restore E-cadherin Expression in Genital and Urinary Cancers

Compound	Description	Cancer	Mechanism and Function	Reference
17-DMCHAG	an Hsp90 inhibitor derived from geldanamycin	PCa	downregulates ZEB1, Snail1, Slug, and vimentin; upregulates E-cadherin; suppresses cell proliferation; induces apoptosis	^33^
α-Solanine	a glycoalkaloid in nightshade (Solanum nigrum Linn)	PCa	elevates E-cadherin, RECK, TIMP-1, and TIMP2 levels; reduces vimentin, MMP-2, MMP-9, and EMMPRIN levels; suppresses the phosphorylation of PI3K, Akt, and ERK; inhibits cell viability and invasion; suppresses the EMT	^22^
Alisertib	a selective aurora kinase A inhibitor	OC	restores the balance between E-cadherin and N-cadherin; inhibits phosphorylation of Aurora kinase A (AURKA); suppresses Akt/mammalian target of rapamycin (mTOR) and p38 pathways; induces G2/M phase arrest, apoptosis, and autophagy; suppresses the EMT	^57^
Antrocin	a sesquiterpene lactone from Antrodia cinnamomea	BCa	promotes the expression of E-cadherin, Fas, DR5, and Bax and activations of caspase-3, -8, and -9; decreases phosphorylation of FAK, paxillin, MMPs, and vimentin; attenuates phosphorylation of ERK and c-Fos; inhibits cell growth, migration, and invasion; induces apoptosis	^45^
Berberine	a plant-derived isoquinoline alkaloid	CC	reduces MMP-2, MMP-9, urokinase-type plasminogen activator (uPA), N-cadherin, vimentin, VEGF, and HIF-1α; impedes activation of NF-κB; inhibits cell proliferation, migration, and invasion; enhances apoptosis; reduces tumor growth	^49^
BMS-345541	a highly selective IKK inhibitor	PCa	downregulates N-cadherin, Snail, Slug, and Twist; upregulates E-cadherin and p-NDRG1; inhibits cell proliferation, invasion, and metastasis; induces apoptosis	^26^
Caffeic acid (CA) and metformin	metformin, anti-diabetes drug; CA, a natural compound	CC	suppresses the expressions of Snail, MMP-9, vimentin, HIF-1α, and CAIX; enhances the expressions of E-cadherin, occuludin, claudin, and TIMP-1; suppresses the motility of cells; inhibits the EMT process	^50^
Carnosol	an analog of resveratrol	OC	restores E-cadherin expression; inhibits cell adhesion, viability, and growth; suppresses EGF-induced EMT	^61^
DZ-50	a lead quinazoline-based Doxazosin derivative	PCa	increases the expression of E-cadherin; decreases the expressions of N-cadherin, talin1, and IGFBP3; inhibits cell invasion; reverses the EMT; impairs tumor growth	^29^
Everolimus	an mTOR inhibitor	RCC	reduces p-FAK, p-Src, vimentin, and RhoA levels; increases E-cadherin expression; suppresses lung metastasis	^40^
Galeterone, VNPT55	galeterone, a proprietary compound; VNPT55, a new analog of galeterone	PCa	downregulates Snail, Slug, N-cadherin, vimentin, MMP-2/9, NF-κB, Twist1, β-catenin, CD44, and Nanog; upregulates E-cadherin; inhibits migration and invasion	^37^
Ginsenoside Rb1	an active component of Panax ginseng	OC	upregulates E-cadherin, downregulates vimentin and miR-25, inhibits cell migration, suppresses the EMT	^56^
Ginsenoside 20(S)-Rg3	an active component of Panax ginseng	OC	reduces the expressions of HIF-1α, Snail, and vimentin; upregulates E-cadherin; inhibits EMT	^55^
HM015K	a novel silybin derivative	OC	upregulates Bax, Bak, cyclin B1, and E-cadherin; downregulates Bcl-2, N-cadherin, c-Myc, DVL3, DVL2, ABCG2, and ABCB1; induces apoptosis	^59^
Honokiol	a natural phenolic compound from Magnolia grandiflora	BCa	induces the expression of E-cadherin; represses the expressions of N-cadherin, SRC-3, MMP-2, and Twist1; suppresses the EMT; inhibits cell migration and invasion	^48^
Kallistain	a plasma protein with anti-inflammatory functions	CC	reduces MMP-2, MMP-9, uPA, N-cadherin, vimentin, VEGF, and HIF-1α; impedes activation of NF-κB; inhibits proliferation, migration, and invasion; enhances apoptosis; reduces tumor growth	^51^
Litchi seed extract	litchi: known as Chinese cherry, a subtropical fruit tree	PCa	upregulates p21, p27, and E-cadherin; decreases the expressions of vimentin, Snail, cyclin, and CDK; suppresses the Akt/GSK-3β-signaling pathway; inhibits cell migration, invasion, and clonogenic growth; induces apoptosis and cell cycle G1/S phase arrest	^27^
Matrine and cisplatin	matrine: a natural compound from the *Sophora* plant genus	BCa	increases ROS, E-cadherin, β-catenin, Bax, and cleaved caspase-3 levels; decreases fibronectin, vimentin, Bcl-2, p-Akt, p-PI3K, VEGFR2, and VEGF; inhibits migration and invasion; induces cell-cycle arrest; promotes apoptosis	^44^
Melatonin	a methoxyindole synthesized and secreted by the pineal gland	OC	decreases the expressions of Ki67, ZEB1, ZEB2, Snail, vimentin, and MMP-9; increases the expression of E-cadherin; inhibits cell proliferation and migration	^62^
Metformin	an anti-diabetes drug	PCa	upregulates E-cadherin; downregulates N-cadherin, vimentin, Twist, Snail1, ZEB1, COX2, PGE2, and p-STAT3; inhibits cell migration and invasion; represses the EMT	24, 25
MLN4924 (pevonedistat)	a suppressor of protein neddylation	RCC	increases the expression of E-cadherin; represses the expressions of vimentin, p21, p27, and Weel; inhibits cell migration and invasion; induces G2/M arrest	^41^
MSKE	muscadine grape skin extract	PCa	decreases the levels of superoxide, STAT-3, and vimentin; increases the expression of E-cadherin; reduces cell migration; abrogates the EMT process	^21^
N-butylidenephthalide (BP)	derived from Radix Angelica Sinensis (Danggui)	BCa	activates caspase-9 and caspase-3, upregulates E-cadherin, downregulates N-cadherin, increases sensitivity to cisplatin, inhibits cell migration, induces cell death and apoptosis, inhibits tumor growth	^43^
NPV-LDE-225 (erimodegib)	a smoothened inhibitor	PCa	promotes the activation of caspase-3 and cleavage of PARP; increases the expressions of Bax, Bak, and E-cadherin; inhibits Bcl-2, Bcl-XL, XIAP, cIAP1, cIAP2, survivin, Gli1, Gli2, Patched-1, Patched-2, Nanog, Oct-4, c-Myc, Sox-2, Bmi-1, N-cadherin, Snail, Slug, and ZEB1; inhibits Shh-signaling pathway; suppresses cell viability and spheroid formation; induces apoptosis; inhibits tumor growth	^20^
Osthole	a monomer extract of a traditional Chinese herb	RCC	increases the expressions of cleaved caspase-3, Bax, and E-cadherin; inhibits Bcl-2, survivin, MMP-2, MMP-9, N-cadherin, vimentin, Smad-3, Snail1, and Twist1; suppresses cell proliferation, invasion, migration, and colony formation	^39^
PMMB232	a shikonin coumarin-carboxylic acid	CC	upregulates E-cadherin and PDH-E1α, downregulates HIF-1α and PDK1, inhibits proliferation, induces apoptosis	^52^
Resveratrol	a natural polyphenilic agent in grape and red wine	OC	restores E-cadherin expression, downregulates hTERT and HIF-1α, inhibits Src phosphorylation, promotes interference of a Src and HIF-1α/hTERT/Slug-signaling pathway, inhibits cell invasion	^60^
Retinamide VNLG-152	a novel retinamide	PCa	suppresses the expressions of N-cadherin, β-catenin, claudin, Slug, Snail, Twist, vimentin, MMP-2, MMP-9, f-AR/AR-V7, MNK1/2, p-EIF4E, PSA, cyclin D1, and Bcl-2; increases the expression of E-cadherin; inhibits tumor growth and the EMT	^36^
Simvastatin	a cholesterol-lowering medication	PCa	reduces N-cadherin and vimentin, increases E-cadherin, inhibits p38 MAPK phosphorylation, suppresses cell migration and invasion, inhibits TGF-β1-induced EMT	^23^
Sophra flavescebs alkaloid (SFA) gels	a traditional Chinese medicine	CC	promotes Bax and E-cadherin expressions; suppresses Bcl-2, cyclin A, and MMP-2 levels; inhibits Akt/mTOR-signaling pathway; restrains cell proliferation and metastasis; induces G2/M arrest and apoptosis	^54^
Sulforaphane (SFN)	abundant in cruciferous vegetables	PCa	upregulates E-cadherin, downregulates CD44v6 and MMP-2, promotes activation of ERK1/2, inhibits cell invasion and migration	^34^
Tanshinone IIA (Tan-IIA)	an extract from Salvia miltiorrhiza	BCa	suppresses MMP-9/-2, N-cadherin, vimentin, Snail, Slug, and CCL2 levels; inhibits phosphorylation of STAT3; upregulates E-cadherin; inhibits cell migration and invasion	^47^
Tetramethypyrazine (TMP)	one active element from the Chinese medicinal herb Chuanxiong	RCC	upregulates NKG2D ligands MICA/B and E-cadherin; downregulates vimentin and fibronectin; inhibits cell viability, proliferation, apoptosis, invasion, and migration	^38^
Tetrandrine	a bisbenzylisoquinoline alkaloid from Stephaniae	BCa	increases E-cadherin; reduces N-cadherin, vimentin, Slug, and Gli-1; impedes metastasis and invasion; reverses the EMT	^46^
Thymoquinone (TQ)	a major ingredient of Nigella sativa	PCa, CC	increases E-cadherin; decreases vimentin, Twist, ZEB1, Slug, TGF-β, Smad2, and Smad3; represses migration, invasion, and metastasis; reverses the EMT	28, 53
Unmodified gold nanoparticles	a single self-therapeutic nanoparticle	OC	upregulates E-cadherin; downregulates Snail, N-cadherin, and vimentin; inhibits the MAPK pathway; represses the EMT; suppresses tumor growth and metastasis	^58^
Withaferin A (WA)	a natural compound	PCa	inhibits the expression of β-catenin, vimentin, Snail, angiogenic marker factor VIII, and retic; upregulates E-cadherin; suppresses Akt signaling; induces cell death	^35^

### Targeting Signaling Pathways to Restore E-cadherin Expression

Some studies in a variety of cancers have documented a tumor-suppressive role of E-cadherin. Moreover, E-cadherin expression is dysregulated due to a host of genetic and epigenetic mechanisms related to cancer development and progression.^18^ Thus, seeking new drugs that act on E-cadherin-related-signaling pathways could be an effective approach to treat human cancers via restoring E-cadherin expression. In the following paragraphs, we discuss some potent therapeutic compounds with relevant concentrations or doses in multiple studies and the potential usage of them for genital and urinary cancer treatments.

### Prostate Cancer (PCa)

PCa is one of the common carcinomas with increasing incidence in the developed world.^19^ Tumor metastasis is the leading cause of mortality and morbidity of PCa patients. Up to now, some anticancer compounds targeting EMT have been designed to treat PCa. Nanta et al.^20^ demonstrated that NVP-LDE-225 (erismodegib) suppressed human PCa stem cell proliferation and EMT via upregulating E-cadherin and diminishing N-cadherin, snail, and ZEB1 by modulating the miR-200 family both *in vitro* and *in vivo*. Moreover, muscadine grape skin extract (MSKE) reversed snail-induced EMT and concomitantly mitigated cell migration in human PCa, due to restoration of E-cadherin expression and decreased vimentin levels.^21^

One group found that α-Solanine, a naturally glycoalkaloid extracted in nightshade (Solanum nigrum Linn), remarkably stimulated E-cadherin expression while it concurrently attenuated vimentin and matrix metalloproteinase (MMP) expression, indicating that it blocked EMT and inhibited the viability and motility of human PCa cells.^22^ Simvastatin^23^ and metformin^24^, ^25^ displayed inhibition of mesenchymal markers such as N-cadherin and vimentin as well as upregulation of E-cadherin in PCa cells, contributing to a blockade of transforming growth factor beta 1 (TGF-β1)-induced EMT. Additionally, BMS-345541, identified as a highly selective IκB kinase (IKK) inhibitor, exhibited an inhibitory effect in PCa cells via phenotypic reversion of the EMT, as evidenced by an increase in E-cadherin and decreases in N-cadherin, snail, slug, and TWIST.^26^

Guo et al.^27^ reported that litchi seed extracts attenuated the migration and invasion of PCa and reversed EMT partially via negative regulation of the Akt/GSK-3β-signaling pathway, which was correlated with upregulation of E-cadherin and β-catenin and downregulation of vimentin and snail. Thymoquinone, a major ingredient of Nigella sativa, provoked E-cadherin expression and simultaneously reduced vimentin and slug expression in PCa cells via the suppression of the TGF-β/Smad2/3-signaling pathway, thereby inhibiting the EMT process.^28^ A novel agent, DZ-50, impaired the invasive properties of PCa cells through the inhibition of IGFBP3 and subsequent conversion of the EMT.^29^

Reportedly, several heat shock protein 90 (Hsp90) inhibitors have elicited anticancer capacity in various preclinical and clinical trials, including geldanamycin.^30^, ^31^ Nevertheless, the liver toxicity of geldanamycin directly impeded its application in patients with PCa.^32^ Hence, a new agent with lower hepatotoxicity and higher effect derived from geldanamycin, 17-DMCHAG, inhibited the proliferation and colony formation of human PCa cell lines and also showed strong anticancer effects in xenograft nude mice via Hsp90 repression, as manifested by the upregulation of E-cadherin and downregulation of vimentin.^33^ Moreover, 3-4,5-dimethylthiazol-2,5-diphenyltetrazolium bromide (MTT) assay revealed that 17-DMCHAG showed high tumor-suppressive activity against PCa cells but lower cytotoxicity against normal prostate cells.^33^

Sulforaphane (SFN), which is abundant in cruciferous vegetables, disrupted invasion via sustained activation of ERK1/2 to the upregulation of E-cadherin and the downregulation of CD44v6 and MMP-2 in human PCa cells.^34^ Specifically, one transgenic mouse model study showed that oral application of withaferin A (WA) abrogated tumorigenesis and progression of PCa, and it facilitated the expression of E-cadherin but diminished the expression of vimentin and snail.^35^ Castration is the standard therapeutic treatment for advanced PCa but with limited benefit, due to the profound relapse and metastasis. Notably, three novel compounds, including the retinamide VNLG-152,^36^ galeterone, and its analog VNPT55,^37^ significantly reduced the migration and invasion in castration-resistant PCa cells via suppression of the EMT.

### Renal Cell Carcinoma (RCC)

RCC is the most common type of kidney cancer in adults and the major cause of mortality in urological cancer. A few compounds have been explored to possess anti-tumor capability for RCC in the last 5 years. Tetramethypyrazine (TMP), one of the active elements derived from the traditional Chinese medicinal herb Chuanxiong, prominently suppressed cell invasion and migration via the obstruction of NKG2D-signaling pathways in RCC.^38^ Molecular mechanisms were further elucidated that TMP inhibited EMT progression, as clarified by the upregulation of E-cadherin and downregulation of vimentin and fibronectin.^38^

In addition, osthole, another monomer extract of a traditional Chinese herb, has been verified as an anticancer compound, because it inhibited the proliferation and colony formation of RCC cell lines due to increased E-cadherin expression and decreases in MMP, N-cadherin, and vimentin expressions.^39^ One group found that everolimus exhibited anti-invasion and anti-migration effects by repressing EMT and focal adhesion kinase (FAK) activity in RCC, both *in vitro* and *in vivo*.^40^ This effect was associated with the upregulation of E-cadherin and downregulation of vimentin.^40^ Neddylation is a post-translational protein modification correlated with carcinogenesis and cancer progression. Hence, MLN4924 (pevonedistat), known as a suppressor of protein neddylation, inhibited growth and survival of human RCC through the activation of E-cadherin and reduction of vimentin.^41^

### Bladder Cancer (BCa)

Although cisplatin is one of the first-line drugs to treat BCa, ineluctable side effects and drug resistance could not be ignored.^42^ It was reported that N-butylidenephthalide (BP), derived from Radix Angelica Sinensis (Danggui), inhibited the migration and invasion of BCa cells, possibly by improving E-cadherin levels and reducing N-cadherin expression.^43^ Importantly, the sensitivity of BCa cells to cisplatin increased dramatically with a combination therapy of BP.^43^ Additionally, matrine administration combined with cisplatin negatively regulated the vascular endothelial growth factor (VEGF)/phosphatidylinositol 3-kinase (PI3K)/Akt-signaling pathway via the upregualtion of E-cadherin and downregulation of vimentin, creating a synergistic efficacy in suppressing growth and metastasis of BCa cells.^44^

Tumor invasion and migration, which are mainly induced by the EMT, cause the most death by BCa. Furthermore, Chiu et al.^45^ found that antrocin, a sesquiterpene lactone isolated from Antrodia cinnamomea, significantly reduced cell growth and metastasis in BCa by modulating some EMT-related genes (upregulation of E-cadherin and downregulation of vimentin). One group demonstrated that tetrandrine impeded the EMT process via the downregulation of Gli-1 expression, as evidenced by an increase in E-cadherin expression and decreases in N-cadherin, vimentin, and slug expressions.^46^ Besides, tanshinone IIA (Tan-IIA) suppressed signal transducers and activators of transcription 3 (STAT3)/CCL2 signaling and resulted in an elevation in E-cadherin levels and reductions in N-cadherin and vimentin, leading to a reversal of the EMT in BCa cells.^47^ Honokiol exhibited an inhibitory effect on BCa cell invasion via blockade of SRC-3 expression and the EMT.^48^ Mechanism dissection revealed that MMP-2 and TWIST1 were downregulated, and E-cadherin expression was restored.^48^

### Cervical Cancer (CC)

Although the human papillomavirus (HPV)-specified vaccine has been increasingly implicated for CC prevention, it is also urgent and essential to identify a new chemotherapy regimen for women diagnosed with metastatic CC.^19^ Several compounds have been investigated to play pleiotropic anti-tumor effects in CC by acting on different molecular targets. For example, berberine reversed TGF-β1-induced EMT, as confirmed by the upregulation of E-cadherin and downregulations of N-cadherin and snail, suppressing metastasis and angiogenesis in human CC cells.^49^

Notably, one group first revealed that caffeic acid (CA) inhibited TGF-β1-triggered EMT mainly via the reductions of vimentin and snail and induction of E-cadherin.^50^ Furthermore, when CA was used together with metformin, compounds displayed a greater suppressive effect on EMT than CA alone. Moreover, kallistatin inversed the EMT process and was accompanied by an increase in E-cadherin expression and decreases in N-cadherin and vimentin expressions through blockade of the nuclear factor κB (NF-κB)-signaling pathway.^51^

A shikonin coumarin-carboxylic acid, PMMB232, was shown to facilitate the expression of E-cadherin protein and curtail the expression of HIF-1α, mediating the apoptosis of HeLa cells.^52^ Similarly, a natural product thymoquinone (TQ) was found to restrain EMT-related transcription factors TWIST1 and ZEB1 and increase E-cadherin expression, thereby retarding migration and invasion of CC cells.^53^ Zhou et al.^54^ had demonstrated that sophra flavescebs alkaloid (SFA) gels, a compound traditional Chinese medicine, suppressed CC cell growth and metastasis and induced apoptosis via the stimulation of E-cadherin and inhibition of MMP-2 and Bcl-2.

### Ovarian Cancer (OC)

The prognosis of patients with OC has remained poor mainly due to aggressive cancer progression. The anti-EMT function and mechanism of ginsenoside 20(S)-Rg3 in OC cells was reported to antagonize hypoxia-induced E-cadherin downregulation and vimentin upregulation through a decrease in HIF-1α.^55^ Subsequently, another pharmacologically active component of Panax ginseng, ginsenoside Rb1, blocked hypoxia-mediated EMT in human OC cells via abrogating the inhibition of miR-25, which was further confirmed by an increase in E-cadherin and a decrease in vimentin.^56^ Alisertib, known as an aurora kinase A inhibitor, suppressed the EMT-like phenotype through restoring E-cadherin, leading to apoptosis and autophagy in human OC cell lines.^57^

It is worth noting that biomedical administrations of nanotechnology have taken tremendous strides. However, the biological characterization and function of unmodified nanoparticles remain unclear. A previous study had recommended that unmodified gold nanoparticles (AuNPs) repressed the proliferation and metastasis of OC cells via inhibition of the mitogen-activated protein kinase (MAPK)-signaling pathway and reversal of the EMT, with concomitant upregulation of E-cadherin and downregulations of snail, N-cadherin, and vimentin.^58^ A novel silybin derivative, HM015K, inversed the metastatic potential of OC cells mainly through significant promotion of E-cadherin expression and mitigation of N-cadherin expression.^59^

Stress hormone norepinephrine (NE) has been thought to be consistent with the acquisition of cancer development, and resveratrol dramatically attenuated NE-induced EMT in OC cells accompanied by the recovery of E-cadherin expression.^60^ Furthermore, carnosol, an analog of resveratrol, had been shown to restore the expression of E-cadherin through suppressing epidermal growth factor (EGF)-induced EMT process in OC cells.^61^ Moreover, melatonin^62^ and selenium-enriched polysaccharides^63^ exhibited a potent inhibitory effect on human OC via hampering the invasion and migration of OC cells. Additionally, these findings were supported by elevating the level of E-cadherin and reducing the expressions of vimentin, ZEB1, and ZEB2.

### Conclusions

In this review, we attempted to summarize the function of E-cadherin and restoration of E-cadherin by natural agents as novel therapeutic strategy for human genital and urinary cancers; however, we could not cite all published reports due to space limitations. We sincerely apologize for those works not cited. In conclusion, E-cadherin has been regarded as having a key role in tumorigenesis and cancer progression. Loss of E-cadherin promoted and regulated multiple signaling pathways that induce the EMT and cancer metastasis. Based on literature reviews in the last 5 years, considerable molecules or agents have been found to reverse the EMT process and retard cancer development by restoring the expression of E-cadherin. However, definitely before any clinical translation, further studies are needed to investigate exact mechanisms and targets of these compounds on anticancer therapies.

E-cadherin is critically involved in the EMT process, which is mainly recognized as an important step for tumor invasion and migration.^64^ Therefore, the EMT plays a key role in cancer progression and metastasis. Since the EMT is a key step facilitating cancer invasion and metastasis, there is an urgent need to develop efficacious and less toxic drugs targeting the EMT for the treatment of human cancers.^64^ Moreover, the EMT is associated with cancer stem cells and drug resistance in human cancers. Thus, targeting the EMT could be helpful for the inhibition of cancer stem cells and overcoming drug resistance.^65^, ^66^ Numerous compounds have been shown to possess anti-tumor activities through the regulation of key molecules in signaling pathways, including E-cadherin. These compounds could be promising new therapeutic agents for the treatment of human cancers. It is important to note that the inhibition of E-cadherin expression is not sufficient per se to induce the EMT, as it has also been described that re-expressing E-cadherin may not be sufficient to induce the mesenchymal-epithelial transition (MET) in cells that are undergoing the EMT.^67^ This suggests that targeting E-cadherin and other EMT molecules could be required to reverse the EMT process.

It is known that chemotherapeutic drugs have unwanted toxicity, such as the cytotoxicity in the gastrointestinal tract. In addition, one drug often targets one molecule in one signaling pathway. However, cancer development and progress are due to deregulation of multiple signaling pathways. Therefore, using one chemical compound does not have any obvious advantages to treat cancer. Due to the non-toxic nature and targeting of multiple signaling pathways by natural agents from dietary sources (known as nutraceuticals), it is a novel strategy to use nutraceuticals for treating human cancer.^68^, ^69^ Although these compounds exhibit their potential to inhibit the development and progression of cancers, there are still several challenges to developing them as therapeutic drugs for cancer patients. For example, poor bioavailability *in vivo* is the main disadvantage of these compounds.^70^ Modification of compound structures has produced actual improvements in bioavailability.^71^ Nanoparticle-based delivery systems could be useful to deliver the agents to targeted organs.^72^ In addition, most studies defined the function of natural agents to restore E-cadherin in cell culture system. Do these compounds possess anticancer activities in different animal models? Thus, further investigations are required to determine the safety and side effects of natural compounds using animal models prior to clinical trials.

Which molecular mechanisms are involved in these compound-regulated E-cadherin expressions? Are these compounds safe for treating human cancer patients without side effects? How do we enhance the delivery efficacy of these compounds to targeted organs? Do cancer patients have better treatment outcomes when using these compounds in combination with chemotherapeutic drugs? Further exploration of the underlying mechanisms of anti-tumor activity by natural compounds could contribute to the future application of these promising agents for the treatment of human cancers in the near future.

## Author Contributions

Y.S., M.Y., and J.Z. searched the literature regarding E-cadherin and compounds in cancer and made the figures and tables. Y.S., Z.W., and X.Z. wrote the manuscript. All authors read and approved the final manuscript.

## Conflicts of Interest

The authors declare no competing interests.
